# Supplementary material for: Heat adaptation of phage T7 under an extended genetic code
Source: Virus Evol. 2021 Dec 1;7(2):veab100. doi: 10.1093/ve/veab100 (PMC8923235; doi:10.1093/ve/veab100)
Supplement: veab100_Supp [file veab100_supp.zip › Sequencing_Analysis.zip!/Sequencing_Analysis/A3/calibration/0.unique_coverage.pdf]

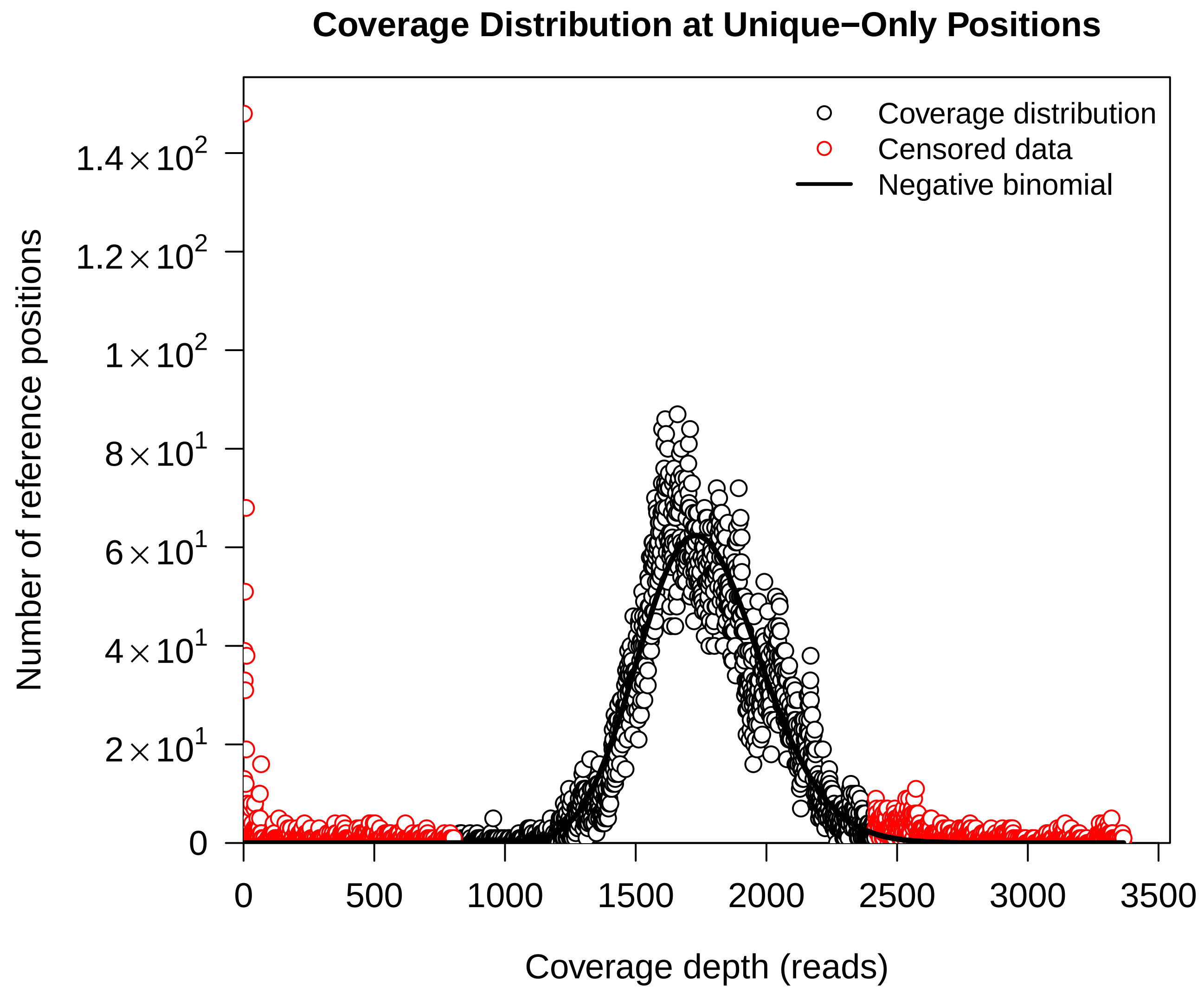

Coverage Distribution at Unique−Only Positions
Coverage distribution
Censored data
Negative binomial
Number of reference positions
1.4×10^2
1.2×10^2
1×10^2
8×10^1
6×10^1
4×10^1
2×10^1
0
0
500
1000
1500
2000
2500
3000
3500
Coverage depth (reads)
